# Analysis of Age, Sex, Lack of Response to Intravenous Immunoglobulin, and Development of Coronary Artery Abnormalities in Children With Kawasaki Disease in Japan

**DOI:** 10.1001/jamanetworkopen.2022.16642

**Published:** 2022-06-13

**Authors:** Nobuhito Takekoshi, Naomi Kitano, Takashi Takeuchi, Tomohiro Suenaga, Nobuyuki Kakimoto, Takayuki Suzuki, Tomoya Tsuchihashi Kada, Shoichi Shibuta, Shinya Tachibana, Yuri Murayama, Hironobu Yamaga, Hiroyuki Suzuki

**Affiliations:** 1 Department of Pediatrics, School of Medicine, Wakayama Medical University, Wakayama, Japan; 2 Division of Pediatrics, Naga Municipal Hospital, Iwade, Japan; 3 Health Administration Center, School of Medicine, Wakayama Medical University, Wakayama, Japan; 4 Division of Pediatrics, Kainan Municipal Hospital, Kainan, Japan; 5 Division of Pediatrics, Kinan Hospital, Tanabe, Japan; 6 Division of Pediatrics, Hashimoto Municipal Hospital, Hashimoto, Japan; 7 Division of Pediatrics, Tsukushi Medical and Welfare Center, Iwade, Japan

## Abstract

**Importance:**

Initial intravenous immunoglobulin (IVIG)–refractory status and prolonged fever are established risk factors for the development of coronary artery abnormalities (CAAs) among patients with acute-phase Kawasaki disease (KD). However, whether different risk factors exist for initial unresponsiveness to IVIG and CAA development remains unclear.

**Objective:**

To evaluate whether different risk factors exist for initial unresponsiveness to IVIG and CAA development among patients with KD (stratified by age at disease onset).

**Design, Setting, and Participants:**

This retrospective cohort study included a consecutive sample of 2414 patients from a database of patients with KD from October 1, 1999, to September 30, 2019. The data were based on annual surveys (response rate, 100%) using hospital medical records across Wakayama Prefecture, Japan. Data were analyzed from March 6 to March 26, 2022.

**Exposures:**

The patient’s age and diagnosis of KD by board-certified pediatricians using the criteria established by the Japan KD Research Committee.

**Main Outcomes and Measures:**

Initial unresponsiveness to IVIG, defined as treatment with optional or advanced therapies, and development of CAAs. Echocardiograms performed 1 month after KD onset using the Japanese Ministry of Health criteria evaluated the presence or absence of CAAs. Odds ratios (ORs) with 95% CIs of patient age at KD onset for unresponsiveness to IVIG and developing CAAs were calculated using multivariable logistic regression models.

**Results:**

A total of 2414 patients (1403 male patients [58.1%]; median age at onset of KD, 25 months [range, 1-212 months]) were included in the study: 550 younger than 12 months, 1342 aged 12 to 47 months, and 522 older than 47 months. A total of 535 patients (22.2%) received optional or advanced treatment and 68 patients (2.8%) developed CAAs 1 month after disease onset. The sex-adjusted OR among patients younger than 12 months for unresponsiveness to IVIG was 0.77 (95% CI, 0.59-0.99) and for development of CAAs was 1.94 (95% CI, 1.07-3.52); among those older than 47 months, the OR for unresponsiveness to IVIG was 1.32 (95% CI, 1.05-1.67) and for development of CAAs was 2.47 (95% CI, 1.39-4.39). After adjusting for IVIG administration, ORs among boys older than 47 months for unresponsiveness to IVIG was 1.14 (95% CI, 0.84-1.56) and for development of CAAs was 2.15 (95% CI, 1.08-4.30); among girls younger than 12 months, the OR for unresponsiveness to IVIG was 1.02 (95% CI, 0.65-1.60) and for development of CAAs was 3.79 (95% CI, 1.21-11.90).

**Conclusions and Relevance:**

The results of this study suggest that risks of unresponsiveness to IVIG and the development of CAAs differ between infants with KD and older patients with KD. Residual risk factors for KD-related CAAs other than initial unresponsiveness to IVIG should be addressed, particularly in infants.

## Key Points

**Question:**

Are age and sex associated with unresponsiveness to intravenous immunoglobulin (IVIG) and the development of coronary artery abnormalities (CAAs) among patients with Kawasaki disease (KD)?

**Findings:**

This cohort study using data from 2414 patients in Japan identified an association between the proportion of patients with initial unresponsiveness to IVIG and increasing age. Differences in the risk factors were observed between initial unresponsiveness to IVIG and the development of CAAs in infants with KD.

**Meaning:**

This study suggests that residual risks other than initial unresponsiveness to IVIG must be addressed to prevent CAA complications among patients with KD, particularly in infants.

## Teaser

This cohort study evaluates whether different risk factors exist for initial unresponsiveness to intravenous immunoglobulin and development of coronary artery abnormalities among patients in Japan with Kawasaki disease.

## Introduction

Kawasaki disease (KD) is a systemic vasculitis that affects mainly children younger than 5 years.^1,2,3^ It is characterized by a prolonged fever that is unresponsive to antibiotics; a polymorphous rash; erythema of the oral mucosa, lips, tongue, palms, and soles; bilateral conjunctival injection; and cervical lymphadenopathy. Kawasaki disease is often complicated by coronary artery abnormalities (CAAs), and approximately 15% to 25% of untreated children develop coronary artery aneurysms.^1,4^ Kawasaki disease is currently the most common cause of pediatric-acquired heart disease in developed countries.^1,5^ The primary goal of treatment for the acute phase of KD is to rapidly inhibit the acute-phase systemic inflammatory response in affected patients and successfully prevent the development of CAAs.

Intravenous immunoglobulin (IVIG) is the primary therapy for KD and has been shown to reduce the incidence of CAAs.^1^ However, approximately 20% of patients with KD do not respond to the initial IVIG treatment and receive additional IVIG treatments and/or other therapies.^1,5,6,7^ Patients with immunoglobulin-refractory KD have a higher risk of developing CAAs.^5^ Therefore, estimating immunoglobulin-refractory KD and initiating the appropriate treatment regimens early has become a focus of clinical trials. In Japan, scoring systems such as the Harada,^8^ Gunma,^9^ Kurume,^10^ and Osaka^11^ scoring systems have been incorporated into treatment algorithms, including the clinical guideline for the medical treatment of acute stage Kawasaki disease.^12,13^ Therefore, in a clinical setting, we assessed the treatment strategy for KD using these scoring systems.

However, nationwide surveys have shown that the incidence of CAA development in Japan has not changed substantially in recent years (2013-2014: CAAs, 2.6%; and giant aneurysms, 0.20%^2^; 2015-2016: CAAs, 2.3%; and giant aneurysms, 0.13%^14^; and 2017-2018: CAAs, 2.6%; and giant aneurysms, 0.11%^15^). Additionally, in clinical practice, some patients with CAAs show resolution of fever with initial IVIG therapy or naturally without treatment.^16,17,18,19,20,21^ Thus, even if effective treatments for immunoglobulin-refractory KD are established, the development of KD-related CAAs cannot always be prevented.

In addition, previous epidemiologic studies have reported that a younger or older age and male sex are associated with the development of CAAs in patients with acute-phase KD.^1,2,14,15,22,23^ A previous study by several authors of the present study also indicated that the male-to-female ratio of patients tended to decrease with increasing age.^24^ Therefore, if patient age is a risk factor for refractory KD or KD-related CAAs, the interaction between patient age and sex should be addressed. However, to our knowledge, few studies have focused on the association of age at the onset of KD and patient sex with refractory KD. The present study aimed to evaluate the difference in the risk associated with patient age in patients with refractory KD and the presence of CAAs; this analysis also incorporated the sex of the patients.

## Methods

This study used a retrospective cohort design to analyze data from patients with KD based on official medical records and prospectively recorded medical information. The study protocol was approved by the ethics committee of Wakayama Medical University. This observational study obtained informed consent from all participants in the form of an opt-out choice, which was mentioned on the Wakayama Medical University website. This report followed the Strengthening the Reporting of Observational Studies in Epidemiology (STROBE) reporting guideline for cohort studies.

### Study Period and Participants

The study was performed in Wakayama Prefecture, Japan. Based on census data, the pediatric population (considered residents younger than 15 years) in this 4726-km^2^ area was 116 412 (59 450 boys and 56 962 girls) in 2015.^25^ In Wakayama Prefecture, the Wakayama Kawasaki Disease Study group, a multicenter clinical and research network for KD, has conducted hospital-based surveys of patients with KD every October since the 1980s at all hospitals with a pediatric department. In these surveys, pediatricians were asked to report all cases of KD based on information from hospital medical records. The data collected from these surveys included patient age (in months) at disease onset, sex, the date of disease onset, the administration of initial IVIG, the use of advanced or optional therapies (eg, additional IVIG, ulinastatin, cyclosporin, corticosteroid pulse therapy, and corticosteroids not using pulse therapy), and coronary artery findings 1 month after KD onset.

We constructed a database of patients with KD across Wakayama Prefecture, Japan, using accumulated information from our annual surveys.^23,24^ During this period, these surveys had a 100% response rate, with no excluded patients and no missing information for the data collected. In this study, 2414 consecutive patients identified between October 1, 1999, and September 30, 2019, were analyzed, and no data were excluded (eFigure in the Supplement). Data were analyzed from March 6 to March 26, 2022.

### Diagnostic Criteria for KD (Inclusion Criteria)

The diagnosis of KD was made based on the clinical criteria from the fourth, fifth, and sixth diagnostic guidelines established by the Japan Kawasaki Disease Research Committee.^26,27,28^ The diagnostic criteria for KD comprise the following 6 main symptoms: a prolonged fever lasting 5 days or more; bilateral nonpurulent conjunctival injection; erythema of the oral mucosa, lips, and tongue; a polymorphous rash; erythematous indurations of the palms and soles; and nonpurulent cervical lymphadenopathy. In the present study, the clinical diagnosis of KD included 2 diagnostic categories: (1) patients who received a diagnosis based on at least 5 of the 6 principal symptoms or 4 of those symptoms when coronary aneurysm or dilatation was observed on a 2-dimensional echocardiograph (considered a complete presentation) and (2) patients who received a diagnosis based on 4 main symptoms without coronary aneurysm or dilatation observed on 2-dimensional echocardiograph or fewer main symptoms with or without coronary aneurysm or dilatation observed on 2-dimensional echocardiograph (considered an incomplete presentation).^2,26,27,28^

### Exposure Variable

The exposure variable was the patients’ age. Patients with KD were diagnosed by board-certified pediatricians based on the diagnostic criteria established by the Japan KD Research Committee.^26,27,28^

### Definition of the Presence of Optional or Additional Therapies (Outcome Variable)

The primary outcome measure was the dichotomous variable of the presence or absence of treatment with optional or additional therapies (ie, ≥1 additional IVIGs and/or ≥1 optional therapies, including ulinastatin, corticosteroids, cyclosporine, infliximab, and plasma exchange). The primary treatment for KD in clinical settings involves oral aspirin and/or initial IVIG therapy, and optional or additional therapies were adopted if the patient remained febrile (≥37.5 °C) at 24 to 36 hours after initiation of the primary therapy.^12,13^ Thus, optional or additional therapies indicate that the patient was refractory to primary therapy, indicating unresponsiveness to IVIG.

### Evaluation of CAAs (Outcome Variable)

Coronary artery abnormalities associated with KD were evaluated using transthoracic 2-dimensional echocardiography 1 month (approximately 30 days) after KD onset. Throughout this study period, clinicians evaluated the presence or absence of CAAs using the following Japanese Ministry of Health (JMH) criteria: CAAs including giant, medium, or small aneurysms and dilatation, which was defined as an internal lumen diameter greater than 3 mm in children younger than 5 years or greater than 4 mm in children aged 5 years or older; an internal diameter of a segment measuring 1.5 times or more that of an adjacent segment; or a clear irregularity in the coronary lumen.^2,14,29,30^

### Statistical Analysis

A total of 2414 consecutive patients with KD and no missing data who were identified between October 1, 1999, and September 30, 2019, were analyzed. No patients were excluded (eFigure in the Supplement). First, we described the characteristics of the study participants. After analyzing patient age distribution, the patient data were divided into 3 categories according to age at disease onset (550 [22.8%] <12 months, 1342 [55.6%] aged 12-47 months, and 522 [21.6%] >47 months). The type of hospital in which patients with acute-phase disease were treated was divided into 3 categories (flagship hospitals, regional core hospitals, and others). The illness day of administration of initial IVIG therapy was divided into 4 categories (<4 days, 4-6 days, >6 days, and missing). The regimen of initial IVIG therapy was divided into 3 categories (2 g/kg/24 hours, others, and none). The study period was divided into 3 categories (October 1999 to September 2006, October 2006 to September 2013, and October 2013 to September 2019) based on our annual surveys. Next, descriptive analyses of the study variables were performed between the groups with the presence or absence of optional or advanced treatment. Statistically significant differences in the categorical variables were tested using the χ^2^ test.

The primary outcome measure was the dichotomous variable of the presence or absence of optional or advanced treatment, which we defined as patients who received optional and/or advanced treatment, including additional IVIG. The odds ratios (ORs) with 95% CIs of patient age at the onset of KD (<12 months and >47 months) for the presence of optional or advanced treatment were calculated with reference to 12- to 47-month-old patients using multivariable logistic regression models adjusted for patient sex (model 1), the study period and the starting illness day of initial IVIG administration (model 2), and the category of the hospital in which patients with acute-phase disease were treated (model 3). The ORs with 95% CIs for the presence of CAAs 1 month after disease onset based on patient age were also calculated using the same models.

For the sensitivity analyses, the patients’ ages at disease onset were divided into 5 categories^24^ (81 [3.4%] <4 months, 405 [16.8%] 4-10 months, 1406 [58.2%] 11-47 months, 430 [17.8%] 48-83 months, and 92 [3.8%] ≥83-212 months) and ORs with 95% CIs were calculated using the same model.

All statistical analyses were performed using SPSS, version 25 (IBM Corp). Statistical significance was defined as a 2-tailed *P* < .05.

## Results

**Figure 1.**
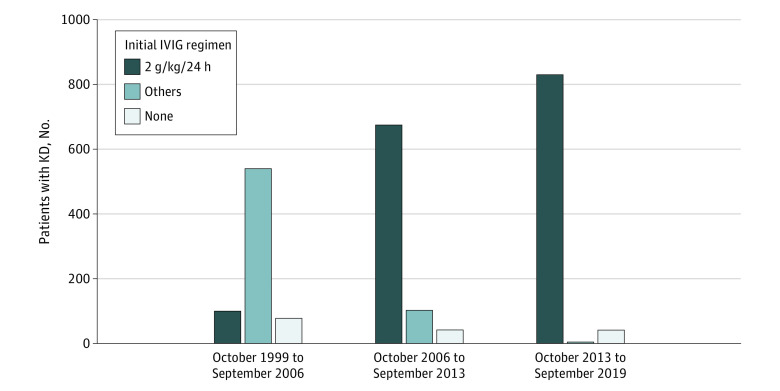
Transition of the Initial Intravenous Immunoglobulin (IVIG) Therapy Regimen Among Patients With Kawasaki Disease (KD) During the Study Period (N = 2414) The study period was divided into 3 categories (October 1999-September 2006, October 2006-September 2013, and October 2013-September 2019) from our annual surveys. In the present study, the initial IVIG therapy regimen converged to 2 g/kg/24 hours.

We analyzed data from 2414 patients (1403 male patients [58.1%]; median age at onset of KD, 25 months [range, 1-212 months]): 550 younger than 12 months, 1342 aged 12 to 47 months, and 522 older than 47 months. The regimen of initial IVIG therapy converged to 2 g/kg/24 hours (Figure 1).

Table 1 shows the characteristics of the study participants at the onset of KD. The proportions of male patients decreased with increasing age (<12 months, 347 of 550 [63.1%]; 12-47 months, 771 of 1342 [57.5%]; and >47 months, 285 of 522 [54.6%]). A total of 535 patients (22.2%) were treated with optional or advanced therapies. An association was found between the proportion of patients treated with optional or advanced therapies and increasing age: 18.2% (100 of 550) of the patients receiving optional or advanced therapies were younger than 12 months, 22.0% (295 of 1342) were aged 12 to 47 months, and 26.8% (140 of 522) were older than 47 months. A total of 68 patients (2.8%) had developed CAAs 1 month after disease onset, with a U-shaped distribution, and the lowest proportion of patients with CAAs was in the 12- to 47-month age group. A total of 360 of 1403 male patients (25.7%) and 175 of 1011 female patients (17.3%) were treated with optional or advanced therapies (*P* < .001) (Table 2). Regarding the primary intensified therapies, our data included 5 patients treated with cyclosporine, but no patients were treated with corticosteroids in this study period.

**Table 1.** Characteristics of the Children Stratified by Patient Age at Onset of KD^a^

Characteristic	Children, No. (%)				*P* value^b^
	Total	Age at onset of KD, mo			
		<12 (n = 550)	12-47 (n = 1342)	>47 (n = 522)	
Sex					
Male	1403 (58.1)	347 (63.1)	771 (57.5)	285 (54.6)	.01
Female	1011 (41.9)	203 (36.9)	571 (42.5)	237 (45.4)	
Study period					
October 1999 to September 2006	718 (29.7)	160 (29.1)	406 (30.3)	152 (29.1)	.95
October 2006 to September 2013	819 (33.9)	191 (34.7)	454 (33.8)	174 (33.3)	
October 2013 to September 2019	877 (36.3)	199 (36.2)	482 (35.9)	196 (37.5)	
Hospital type in which patients with acute-phase disease were treated					
Flagship hospital	1483 (61.3)	332 (60.4)	824 (61.4)	327 (62.6)	.15
Regional core hospital	799 (33.1)	198 (36.0)	435 (32.4)	166 (31.8)	
Other	132 (5.5)	20 (3.6)	83 (6.2)	29 (5.6)	
Medical treatment					
Primary treatment					
Oral aspirin	2377 (98.5)	538 (97.8)	1321 (98.4)	518 (99.2)	.17
IVIG therapy	2253 (93.3)	504 (91.6)	1266 (94.3)	483 (92.5)	.07
Illness day of initial IVIG treatment					
<4 d	132 (5.5)	41 (7.5)	77 (5.7)	14 (2.7)	<.001
4-6 d	1894 (78.5)	429 (78.0)	1066 (79.4)	399 (76.4)	
>6 d	227 (9.4)	34 (6.2)	123 (9.2)	70 (13.4)	
Missing	161 (6.7)	46 (8.4)	76 (5.7)	39 (7.5)	
Regimen of initial IVIG therapy					
2 g/kg/24 h	1608 (66.6)	359 (65.3)	896 (66.8)	353 (67.6)	.19
Others	645 (26.7)	145 (26.4)	370 (27.6)	130 (24.9)	
None	161 (6.7)	46 (8.4)	76 (5.7)	39 (7.5)	
Optional or additional therapies	535 (22.2)	100 (18.2)	295 (22.0)	140 (26.8)	.003
Additional IVIG	508 (21.0)	89 (16.2)	285 (21.2)	134 (25.7)	.001
Ulinastatin	102 (4.2)	22 (4.0)	44 (3.3)	36 (6.9)	.002
Corticosteroid pulse	29 (1.2)	8 (1.5)	8 (0.6)	13 (2.5)	.003
Corticosteroid without pulse	19 (0.8)	4 (0.7)	6 (0.4)	9 (1.7)	.02
Cyclosporine	130 (5.4)	20 (3.6)	71 (5.3)	39 (7.5)	.02
Infliximab	12 (0.5)	0	5 (0.4)	7 (1.3)	.005
Coronary artery abnormalities	68 (2.8)	20 (3.6)	25 (1.9)	23 (4.4)	.005
Male	47 (3.4)	12 (3.5)	20 (2.6)	15 (5.3)	.10
Female	21 (2.1)	8 (3.9)	5 (0.9)	8 (3.4)	.009

Abbreviations: IVIG, intravenous immunoglobulin; KD, Kawasaki disease.

^a^ All the participants were stratified by patient age at the onset of Kawasaki disease (N = 2414).

^b^ Obtained from the χ^2^ test or the Fisher exact test.

**Table 2.** Characteristics of the Children Stratified by the Presence or Absence of Treatment With Optional or Advanced Therapies

Characteristic	Optional or advanced treatment, No. (%)		*P* value^a^
	Yes (n = 535)	No (n = 1879)	
Sex			
Male	360 (67.3)	1043 (55.5)	<.001
Female	175 (32.7)	836 (44.5)	
Age at onset of KD, mo			
<12	100 (18.7)	450 (23.9)	.003
12-47	295 (55.1)	1047 (55.7)	
>47	140 (26.2)	382 (20.3)	
Study period			
October 1999 to September 2006	132 (24.7)	586 (31.2)	.001
October 2006 to September 2013	175 (32.7)	644 (34.3)	
October 2013 to September 2019	228 (42.6)	649 (34.5)	
Hospital type in which patients with acute-phase disease were treated			
Flagship hospital	389 (72.7)	1094 (58.2)	<.001
Regional core hospital	125 (23.4)	674 (35.9)	
Other	21 (3.9)	111 (5.9)	
Primary therapy			
Oral aspirin	530 (99.1)	1847 (98.3)	.24
IVIG	530 (99.1)	1723 (91.7)	<.001
2 g/kg/24 h	410 (76.6)	1198 (63.8)	.001
Others	120 (22.4)	525 (27.9)	
None	5 (3.1)	156 (8.3)	
Illness day of initial IVIG treatment			
<4 d	40 (7.5)	92 (4.9)	<.001
4-6 d	455 (85.0)	1439 (76.6)	
>6 d	35 (6.5)	192 (10.2)	
Missing	5 (0.9)	156 (8.3)	
Coronary artery abnormalities			
Overall	47 (8.8)	21 (1.1)	<.001
Male	33 (9.2)	14 (1.3)	<.001
Female	14 (8.0)	7 (0.8)	<.001

Abbreviations: IVIG, intravenous immunoglobulin; KD, Kawasaki disease.

^a^ Obtained from the χ^2^ test or the Fisher exact test.

**Figure 2.**
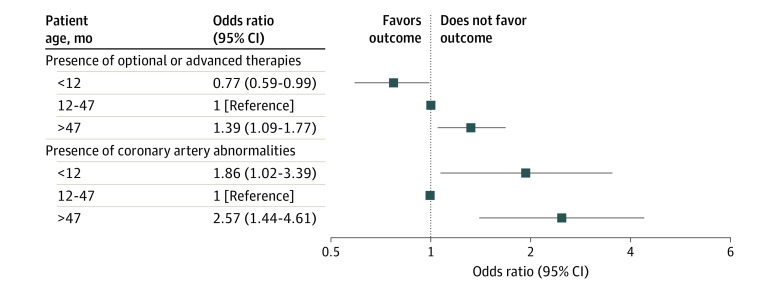
Odds Ratios of Patient Age at the Onset of Kawasaki Disease for the Presence of Optional and Advanced Therapies and Developing Coronary Artery Abnormalities Adjusted for Potential Confounders (N = 2414) The association between patient age group and the 2 outcomes included the following: the adjusted ORs of patient age for the presence of optional or advanced therapies were 0.77 (95% CI, 0.59-0.99) among patients younger than 12 months and 1.39 (95% CI, 1.09-1.77) among those older than 47 months (reference: 12- to 47-month age group), while the adjusted ORs of patient age for the presence of CAAs were 1.86 (95% CI, 1.02-3.39) among patients younger than 12 months and 2.57 (95% CI, 1.44-4.61) among those older than 47 months (reference: 12- to 47-month age group).

Table 3 presents data for patient age group and the 2 outcomes. The sex-adjusted OR among patients younger than 12 months for treatment with optional or advanced therapies was 0.77 (95% CI, 0.59-0.99) and for development of CAAs was 1.94 (95% CI, 1.07-3.52); among those older than 47 months, the OR for treatment with optional or advanced therapies was 1.32 (95% CI, 1.05-1.67) and for development of CAAs was 2.47 (95% CI, 1.39-4.39). The ORs of patient age for the presence of optional or advanced therapies after adjustment for all potential confounders were 0.77 (95% CI, 0.59-0.99) among patients younger than 12 months and 1.39 (95% CI, 1.09-1.77) among those older than 47 months (reference: 12- to 47-month age group), while the ORs for the presence of CAAs after adjustment for all potential confounders were 1.86 (95% CI, 1.02-3.39) among patients younger than 12 months and 2.57 (95% CI, 1.44-4.61) among those older than 47 months (reference: 12- to 47-month age group) (Figure 2). Among male patients, the ORs for the presence of optional or advanced therapies after adjustment for all potential confounders were 0.66 (95% CI, 0.48-0.91) among boys younger than 12 months and 1.14 (95% CI, 0.84-1.56) among boys older than 47 months; ORs for the presence of CAAs after adjustment for all potential confounders were 1.32 (95% CI, 0.64-2.76) among boys younger than 12 months and 2.15 (95% CI, 1.08-4.30) among boys older than 47 months (eTable 1 in the Supplement). Among female patients, the ORs for the presence of optional or advanced therapies after adjustment for all potential confounders were 1.02 (95% CI, 0.65-1.60) among girls younger than 12 months and 1.96 (95% CI, 1.32-2.90) among girls older than 47 months; ORs for the presence of CAAs after adjustment for all potential confounders were 3.79 (95% CI, 1.21-11.90) among girls younger than 12 months and 4.16 (95% CI, 1.31-13.21) among girls older than 47 months.

**Table 3.** Data on the Presence of Treatment With Optional or Advanced Therapies and the Development of CAAs at 1 Month After KD Onset Stratified by 3 Categories of Patient Age at Onset of KD

Outcome^a^	Aged <12 mo		Aged 12-47 mo		Aged >47 mo	
	Population at risk, No. with/No. without	OR (95% CI)	Population at risk, No. with/No. without	OR (95% CI)	Population at risk, No. with/No. without	OR (95% CI)
Incidence of treatment with optional or advanced therapies						
Model 1	100/450	0.77 (0.59-0.99)	295/1047	1 [Reference]	140/382	1.32 (1.05-1.67)
Model 2	100/450	0.77 (0.59-0.99)	295/1047	1 [Reference]	140/382	1.40 (1.10-1.77)
Model 3	100/450	0.77 (0.59-0.99)	295/1047	1 [Reference]	140/382	1.39 (1.09-1.77)
Incidence of coronary artery abnormalities						
Model 1	20/530	1.94 (1.07-3.52)	25/1317	1 [Reference]	23/499	2.47 (1.39-4.39)
Model 2	20/530	1.87 (1.03-3.41)	25/1317	1 [Reference]	23/499	2.61 (1.46-4.66)
Model 3	20/530	1.86 (1.02-3.39)	25/1317	1 [Reference]	23/499	2.57 (1.44-4.61)

Abbreviations: CAAs, coronary arterial abnormalities; KD, Kawasaki disease; OR, odds ratio.

^a^ Model 1: adjusted for patient sex. Model 2: model 1 plus adjusted for the study period and starting illness day of intravenous immunoglobulin administration. Model 3: model 2 plus adjusted for the 3 categories of hospital type in which patients with acute-phase disease were treated.

The results of the sensitivity analyses among the 5 categories of patient ages were consistent: the OR of patient age for the presence of optional or advanced therapies after adjustment for potential confounders was 0.70 (95% CI, 0.52-0.94) among patients aged 4 to 10 months and 1.34 (95% CI, 1.04-1.73) among those aged 48 to 83 months (reference: 11-month to 47-month age group), while the OR for the presence of CAAs was 2.04 (95% CI, 1.06-3.93) among patients aged 4 to 10 months and 2.39 (95% CI, 1.27-4.49) among patients aged 48 to 83 months (reference: 11-month to 47-month age group) (eTable 2 in the Supplement).

## Discussion

Using a database consisting of consecutive patients with KD from a specific area in Japan over 20 years, this study found that the incidence rate of unresponsiveness to IVIG and the development of CAAs might differ among patients younger than 12 months. Our findings suggest that risk factors other than unresponsiveness to IVIG may be associated with the development of CAAs among patients younger than 12 months.

According to the 2020 edition of the Japanese guidelines on acute-stage Kawasaki disease treatment,^13^ intensified primary therapy, such as IVIG plus corticosteroids^31^ or IVIG plus cyclosporine,^32^ should be administered to patients who are estimated by scoring systems to have unresponsiveness to IVIG to prevent the development of CAAs. However, there is a potential problem with the stratification strategy using the following existing scoring systems^13^: the applied scoring systems were designed to estimate which patients would have initial resistance to IVIG but were not designed to estimate and distinguish which patients would have CAAs. Because these scoring systems were generated from individual study populations in a specific area, the outcome variables of these studies were refractory to initial IVIG treatment.^9,10,11^

The findings of this study suggest that a focus on patient age incorporated with patient sex, particularly in infants with KD, is warranted to prevent the development of CAAs in these patients. Although we have little evidence to explain why infants with KD are so vulnerable to CAAs despite not requiring optional or advanced therapies (because of the short duration of fever), we propose several possibilities. First, the immune responses to the triggers of KD in infants may be weak. Thus, these triggers may not allow infants to easily develop systemic inflammatory chain reactions. However, CAAs may develop owing to persistent, localized inflammation in the coronary arterial walls. Therefore, fever, an indicator of systemic inflammation, may be insufficient to induce this type of inflammation in infants with KD. Second, the coronary arteries of infants may be immature and vulnerable. A previous optical coherence tomography study revealed that most CAA lesions had developed from tunica media disruptions.^33^ The tunica media of infants may not be sufficiently strong to resist the damage associated with KD vasculitis, and tunica media disruptions may develop easily. Third, the triggers of KD may be different among different age groups, particularly between infants and children older than 47 months. Some previous studies reported the seasonality of KD occurrence^2,3,23,34,35,36^ and indicated an association between patient age and the onset of KD.^23,35^ The present findings suggest that infant girls and boys older than 47 months, whose age and sex groups have a relatively low incidence of KD onset,^24^ still developed CAAs despite having a lower incidence of additional or optional treatment. Taken together, these findings show that the combined associations of patient age and sex with the development of CAAs warrant further investigation.

### Limitations

This study has some limitations, including those inherent to retrospective studies. In addition, the study population was limited to one geographical area. This study examined data from consecutive patients collated over a 20-year period in one prefecture in Japan (similar to a state in the US); therefore, the findings were based on the data from a study population comprising Japanese patients with KD and may not be generalizable to populations with different racial or ethnic backgrounds. However, the incidence of KD in Japan is high^2,3^; thus, the findings from this study might be used as a representative or reference sample. Second, proving that the present study was based on all patients in the survey area is difficult. Although the possibility of missed patients (eg, those transitioning between prefectures) remains, to our knowledge, all patients with KD who lived in Wakayama Prefecture in the study period were reviewed. Third, 3 or more different regimens of primary therapies were used because the observation period was long (20 years), and the standard primary therapy, particularly the initial dose of IVIG, changed. Therefore, we defined unresponsiveness to IVIG as the presence or absence of treatment with optional or advanced therapies. Fourth, we were unable to use *z* scores but did use the JMH criteria to evaluate CAAs because we collected coronary arterial diameter data from the questionnaires in each annual survey (1999-2019). However, the *z* score criteria of CAAs are stricter than the JMH criteria for young children.^37^ Thus, the incidence of CAA development evaluated using the JMH criteria may be underestimated in younger children. The difference in risk factors between initial unresponsiveness to IVIG and CAA development may be more pronounced, particularly in infants.

## Conclusions

This cohort study identified a difference in risk factors between initial unresponsiveness to IVIG and the development of CAAs among infant patients with KD. Among vulnerable patients with KD, latent risk factors not associated with being refractory to initial IVIG treatment may exist for developing CAAs. Therefore, the residual risks and the initial unresponsiveness to IVIG must be addressed to prevent CAA complications among patients with KD.

## Notes

Supplement. **eTable 1.** Data on the Presence of Treatment With Optional or Advanced Therapies and Development of CAAs at 1 Month After KD Onset, Stratified by Sex, Boys (n=1,403) and Girls (n=1,101) After Stratification Into 3 Categories of Patients' Age at Onset of KD **eTable 2.** Data on the Presence of Treatment With Optional or Advanced Therapies and Development of CAAs at 1 Month After KD Onset (n=2,414) After Stratification Into 5 Categories of Patients' Age at Onset of KD **eFigure.** Flow Chart of the Study Participants
