# Supplementary material for: Analysis of Age, Sex, Lack of Response to Intravenous Immunoglobulin, and Development of Coronary Artery Abnormalities in Children With Kawasaki Disease in Japan
Source: JAMA Netw Open. 2022 Jun 13;5(6):e2216642. doi: 10.1001/jamanetworkopen.2022.16642 (PMC9194667; doi:10.1001/jamanetworkopen.2022.16642)
Supplement: Supplement. — eTable 1. Data on the Presence of Treatment With Optional or Advanced Therapies and Development of CAAs at 1 Month After KD Onset, Stratified by Sex, Boys (n=1,403) and Girls (n=1,101) After Stratification Into 3 Categories of Patients' Age at Onset of KD eTable 2. Data on the Presence of Treatment With Optional or Advanced Therapies and Development of CAAs at 1 Month After KD Onset (n=2,414) After Stratification Into 5 Categories of Patients' Age at Onset of KD eFigure. Flow Chart of the Study Participants [file jamanetwopen-e2216642-s001.pdf]

Supplementary Online Content

Takekoshi N, Kitano N, Takeuchi T, et al. Analysis of age, sex, lack of response to intravenous immunoglobulin, and development of coronary artery abnormalities in children with Kawasaki disease in Japan. *JAMA Netw Open.* 2022;5(6):e2216642. doi:10.1001/jamanetworkopen.2022.16642

eTable 1. Data on the Presence of Treatment With Optional or Advanced Therapies and Development of CAAs at 1 Month After KD Onset, Stratified by Sex, Boys (n=1,403) and Girls (n=1,101) After Stratification Into 3 Categories of Patients' Age at Onset of KD
eTable 2. Data on the Presence of Treatment With Optional or Advanced Therapies and Development of CAAs at 1 Month After KD Onset (n=2,414) After Stratification Into 5 Categories of Patients' Age at Onset of KD
eFigure. Flow Chart of the Study Participants

This supplementary material has been provided by the authors to give readers additional information about their work.

© 2022 Takekoshi N et al. *JAMA Network Open.*

eTable 1. Data on the Presence of Treatment With Optional or Advanced Therapies and Development of CAAs at 1 Month After KD Onset, Stratified by Sex, Boys (n=1,403) and Girls (n=1,101) After Stratification Into 3 Categories of Patients' Age at Onset of KD

**Boys (n=1,403)**																	
	<12 months of age						12-47 months of age						>47 months of age				
	Population at risk			OR	95% CI		Population at risk			OR			Population at risk			OR	
	with/without						with/without				95% CI		with/without				95% CI
Presence of optional/advanced therapies	68	/	279	0.66	(0.48, 0.90)		208	/	563	Univariate	reference		84	/	201	1.13	(0.84, 1.53)
				0.65	(0.48, 0.89)					Model 1	reference					1.15	(0.85, 1.56)
				0.65	(0.48, 0.89)					Model 2	reference					1.15	(0.85, 1.55)
				0.66	(0.48, 0.91)					Model 3	reference					1.14	(0.84, 1.56)
Presence of coronary artery abnormalities	12	/	335	1.35	(0.65, 2.78)		20	/	751	Univariate	reference		15	/	270	2.09	(1.05, 4.13)
				1.31	(0.63, 2.71)					Model 1	reference					2.11	(1.06, 4.19)
				1.31	(0.63, 2.72)					Model 2	reference					2.20	(1.10, 4.38)
				1.32	(0.64, 2.76)					Model 3	reference					2.15	(1.08, 4.30)

© 2022 Takekoshi N et al. *JAMA Network Open.*

**Girls (n=1,011)**															
	<12 months of age					12-47 months of age					>47 months of age				
	Population at risk			OR	95% CI	Population at risk			OR		Population at risk			OR	
	with/without					with/without				95% CI	with/without				95% CI
Presence of optional/advanced therapies	32	/	171	1.04	(0.67, 1.62)	87	/	484	Univariate	reference	56	/	181	1.72	(1.18, 2.51)
				1.06	(0.68, 1.65)				Model 1	reference				2.01	(1.36, 2.95)
				1.06	(0.68, 1.66)				Model 2	reference				1.99	(1.35, 2.93)
				1.02	(0.65, 1.60)				Model 3	reference				1.96	(1.32, 2.90)
Presence of coronary artery abnormalities	8	/	195	4.64	(1.50, 14.36)	5	/	566	Univariate	reference	8	/	229	3.96	(1.28, 12.22)
				4.00	(1.28, 12.52)				Model 1	reference				4.12	(1.31, 12.99)
				4.00	(1.28, 12.52)				Model 2	reference				4.13	(1.31, 13.02)
				3.79	(1.21, 11.90)				Model 3	reference				4.16	(1.31, 13.21)

Model 1: Adjusted for starting illness day of administration of IVIG.

Model 2: Model 1 + adjusted for the study period.

Model 3: Model 2 + adjusted for 3-category hospital type in which acute-phase patients were treated.

© 2022 Takekoshi N et al. *JAMA Network Open.*

eTable 2. Data on the Presence of Treatment With Optional or Advanced Therapies and Development of CAAs at 1 Month After KD Onset (n=2,414) After Stratification Into 5 Categories of Patients' Age at Onset of KD

	**<4 months of age**			**4-10 months of age**			**11-47 months of age**			**48-83 months of age**			**≥84 months of age**		
	Population at risk with/without	OR	95% CI	Population at risk with/without	OR	95% CI	Population at risk with/without	OR	95% CI	Population at risk with/without	OR	95% CI	Population at risk with/without	OR	95% CI
**Presence of optional/ advanced therapies**	16 / 65	0.87	(0.50, 1.53)	69 / 336	0.73	(0.54, 0.97)	310 / 1096	Univariate	reference	116 / 314	1.31	(1.02, 1.67)	24 / 68	1.25	(0.77, 2.02)
		0.83	(0.47, 1.46)		0.70	(0.53, 0.94)		Model 1	reference		1.32	(1.03, 1.69)		1.34	(0.82, 2.18)
		0.76	(0.43, 1.34)		0.70	(0.52, 0.94)		Model 2	reference		1.36	(1.06, 1.75)		1.57	(0.95, 2.58)
		0.77	(0.44, 1.37)		0.70	(0.52, 0.93)		Model 3	reference		1.35	(1.04, 1.73)		1.60	(0.97, 2.64)

© 2022 Takekoshi N et al. *JAMA Network Open.*

				0.74	(0.42, 1.32)					0.70	(0.52, 0.94)					Model 4	reference					1.34	(1.04, 1.73)					1.56	(0.94, 2.58)
**Presence of coronary artery abnormalities**	5	/	76	3.63	(1.35, 9.76)		15	/	390	2.13	(1.11, 4.07)		25	/	1381	Univariate	reference		17	/	413	2.27	(1.22, 4.25)		6	/	86	3.85	(1.54, 9.64)
				3.49	(1.30, 9.39)					2.07	(1.08, 3.97)					Model 1	reference					2.29	(1.22, 4.28)					4.12	(1.64, 10.35)
				3.29	(1.22, 8.89)					1.99	(1.03, 3.81)					Model 2	reference					2.34	(1.25, 4.39)					4.63	(1.82, 11.82)
				3.12	(1.15, 8.45)					2.05	(1.07, 3.94)					Model 3	reference					2.41	(1.28, 4.52)					4.54	(1.78, 11.59)
				3.08	(1.13, 8.39)					2.04	(1.06, 3.93)					Model 4	reference					2.39	(1.27, 4.49)					4.39	(1.71, 11.24)

Model 1: Adjusted for patient sex.

Model 2: Model 1 + adjusted for starting illness day of administration of IVIG.

Model 3: Model 2 + adjusted for the study period.

Model 4: Model 3 + adjusted for the 3-category hospital type in which acute-phase patients were treated.

OR, odds ratio; CI confidence interval.

© 2022 Takekoshi N et al. *JAMA Network Open.*

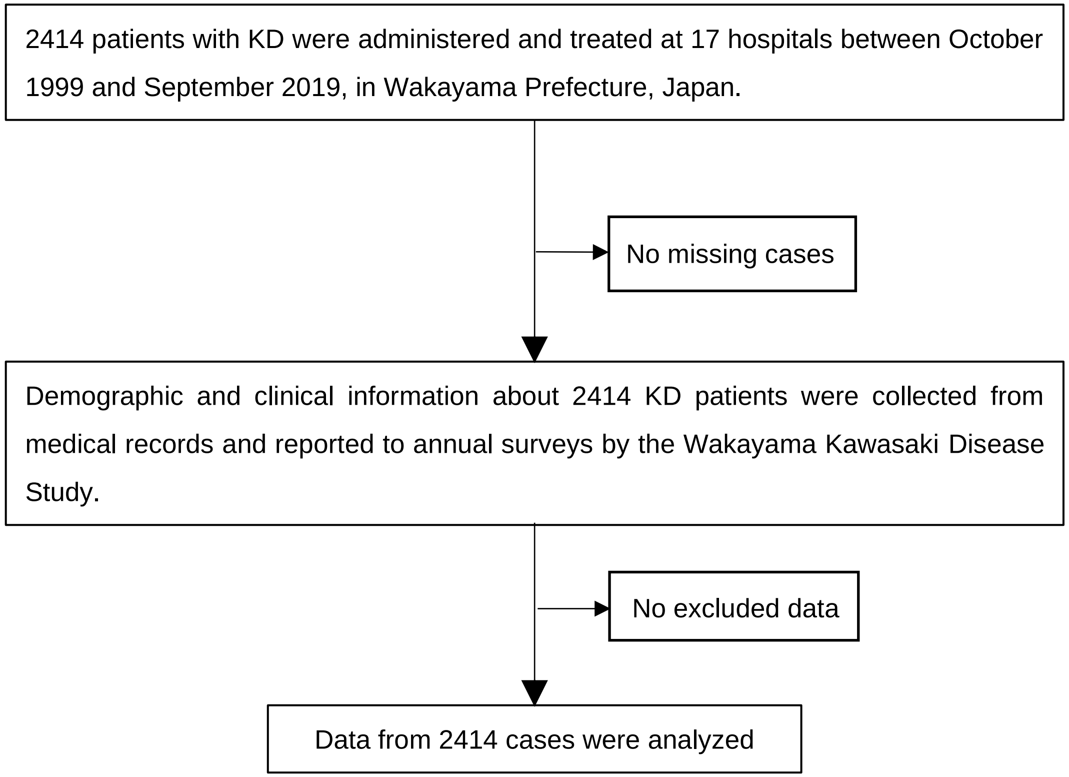

eFigure. Flow Chart of the Study Participants

© 2022 Takekoshi N et al. *JAMA Network Open.*
